# Shikimic Acid Promotes Oligodendrocyte Precursor Cell Differentiation and Accelerates Remyelination in Mice

**DOI:** 10.1007/s12264-018-0322-7

**Published:** 2019-01-25

**Authors:** Fengfeng Lu, Dou Yin, Yingyan Pu, Weili Liu, Zhenghao Li, Qi Shao, Cheng He, Li Cao

**Affiliations:** 1 Institute of Physical Science and Information Technology, Anhui University, Hefei, 230601 China; 2 Institute of Neuroscience, Key Laboratory of Molecular Neurobiology of The Ministry of Education, and The Collaborative Innovation Center for Brain Science, Second Military Medical University, Shanghai, 200433 China

**Keywords:** Shikimic acid, Oligodendrocyte precursor cells, Demyelination, Remyelination

## Abstract

The obstacle to successful remyelination in demyelinating diseases, such as multiple sclerosis, mainly lies in the inability of oligodendrocyte precursor cells (OPCs) to differentiate, since OPCs and oligodendrocyte-lineage cells that are unable to fully differentiate are found in the areas of demyelination. Thus, promoting the differentiation of OPCs is vital for the treatment of demyelinating diseases. Shikimic acid (SA) is mainly derived from star anise, and is reported to have anti-influenza, anti-oxidation, and anti-tumor effects. In the present study, we found that SA significantly promoted the differentiation of cultured rat OPCs without affecting their proliferation and apoptosis. In mice, SA exerted therapeutic effects on experimental autoimmune encephalomyelitis (EAE), such as alleviating clinical EAE scores, inhibiting inflammation, and reducing demyelination in the CNS. SA also promoted the differentiation of OPCs as well as their remyelination after lysolecithin-induced demyelination. Furthermore, we showed that the promotion effect of SA on OPC differentiation was associated with the up-regulation of phosphorylated mTOR. Taken together, our results demonstrated that SA could act as a potential drug candidate for the treatment of demyelinating diseases.

## Introduction

Oligodendrocyte precursor cells (OPCs) are widely distributed in the adult central nervous system (CNS). They originate from neural stem cells that line ventricles in discrete regions throughout the brain and spinal cord during embryonic development, then proliferate and migrate through the white matter, and finally differentiate into myelinating oligodendrocytes (OLs) after birth [1]. Mature OLs wrap axons in layers to form the myelin sheath that protects axons, facilitates the rapid conduction of action potentials, and electrically insulates fibers from one another [2, 3]. Damage to OLs caused by ischemia, hypoxia, or inflammation can lead to demyelination, which not only influences the transmission of neural signals, but also results in irreversible axonal degeneration. In most cases, OPCs rapidly cluster at lesions after demyelination to proliferate and differentiate into mature OLs, form new myelin sheaths, and wrap exposed axons to achieve functional restoration [4]. However, remyelination only occurs at an early stage but fails at later stages in many demyelinating diseases such as multiple sclerosis (MS) [3, 5]. Such a failure of remyelination is mainly attributable to the inability of OPCs to differentiate and mature in demyelinating lesions [6]. Therefore, promoting the differentiation and maturation of OPCs is crucial to the treatment of these demyelinating diseases.

Shikimic acid (SA), a hydrogenated metabolite of the shikimate pathway, is a precursor for the synthesis of aromatic compounds such as cinnamic acid, flavonoids, alkaloids, anthocyanins, indoles, flavones, alcohol and tannins in plants and microorganisms [7–9]. It is also known as the primary base material for the synthesis of the neuraminidase inhibitor Oseltamivir (Tamiflu®), which is commonly used to treat the H5N1 and A/H1N1 strains of influenza [10]. Apart from that, researchers have also found that it has anti-cancer effects [11]. Administration of SA at 200 mg/kg can decrease the expression of pro-inflammatory cytokines induced by lipopolysaccharide and reduce mechanical hyperalgesia in mice [12]. In addition, SA and its derivative, 3,4-oxo-isopropylidene-shikimic acid, can inhibit platelet aggregation and arterial, venous and cerebral thrombosis as well as promote recovery from ischemic injury [13, 14]. However, the effects of SA on CNS demyelinating diseases are not clear. In this study, we demonstrated that SA can promote the differentiation and maturation of OPCs and accelerate remyelination, suggesting a potential application in the clinical treatment of demyelinating diseases.

## Materials and Methods

### Animals and Shikimic Acid

C57BL/6 J mice were purchased from Shanghai SLAC Laboratory Animal Co., Ltd (Shanghai, China), maintained under specific pathogen-free conditions, and used at 6 weeks–8 weeks of age. All animal experiments were performed in adherence with the National Institutes of Health Guidelines on the Use of Laboratory Animals and approved by the Second Military Medical University Committee on Animal Care.

SA was from Sigma-Aldrich (St. Louis, MO), rapidly dissolved in phosphate-buffered saline (PBS) at 100 mg/mL as stock solution (100 ×), and stored in the dark at 4°C. SA was freshly prepared before use.

### *L*-α-lysophosphatidylcholine (LPC) Induced Focal Demyelination in the Dorsal Spinal Cord

Adult (8 weeks–10 weeks old) female C57BL/6 mice were anesthetized by intraperitoneal injection of 3.6% chloral hydrate, and kept on an electric heating pad during the manipulative procedure. Focal demyelination in dorsal spinal cord was induced by LPC (62962, Sigma-Aldrich) as described previously [15, 16]. Briefly, 1 μL of LPC (0.1% in saline) was injected into the dorsal column at the T11–T12 vertebrae with a micromanipulator. Three days after injection, SA and vehicle were intraperitoneally administered. Mice were anesthetized and sacrificed at 7 days and 14 days after LPC injection, then the spinal cord containing the injection lesion was collected and cut into serial paraffin sections. The demyelinated lesion volume was calculated based on the equation: *V* = ∑ demyelinated lesion area × thickness of section [17].

### Experimental Autoimmune Encephalomyelitis (EAE) Model

The EAE model was induced with myelin oligodendrocyte glycoprotein (MOG_35–55_) as previously described [18]. Briefly, female C57BL/6 mice (7 weeks–8 weeks old) were subcutaneously injected with 200 μL of emulsified liquid (mixing ratio, 1:1) consisting of MOG_35–55_ (GL Biochem, Shanghai, China) dissolved in 1 × PBS at 1 mg/mL and heat-killed *Mycobacterium tuberculosis* (H37Ra strain, Difco, Detroit, MI) mixed evenly in incomplete Freund’s adjuvant (Sigma-Aldrich) at 5 mg/mL. Injections were made at 3 points on the back. The day of injection was recorded as 0 day post-injection (dpi). Pertussis toxin (100 ×) (516561, Calbiochem-EMD Chemicals, San Diego, CA) was dissolved in 1 × PBS and administered intraperitoneally at 0 dpi and 2 dpi. SA was injected intraperitoneally at 15 dpi. Clinical EAE scores were graded daily in a blind manner as follows: 0, no observable symptoms; 1, limp tail; 2, limp tail and partial limb weakness; 3, one hindlimb paralyzed; 4, both hindlimbs paralyzed; 5, moribund or dead.

### Primary Oligodendrocyte Progenitor Cell Culture

OPCs were cultured and purified as described previously [19, 20]. Briefly, mixed glial cells were harvested from P0 rat cortex and cultured in Dulbecco’s modified Eagle’s medium with 10% fetal bovine serum for 10 days at 37°C in a 5% CO_2_ incubator. The medium was changed every 3 days. For purification, the flasks were first shaken at 180 rpm for 1 h to remove microglia and at 200 rpm for 16 h with freshly-changed medium at 37°C to collect OPCs. The collected cells were allowed to adhere to uncoated plates for 0.5 h twice to remove contaminating cells. The purified OPCs were collected by gently shaking the plate and seeding them at 5,000 cells/cm^2^–50,000 cells/cm^2^ on coverslips that had been coated with poly-*D*-lysine the previous day. Finally, OPCs were cultured in Neurobasal medium supplemented with 2% B27 for differentiation.

### Immunocytofluorescence Staining

Cells on coverslips were fixed in 4% paraformaldehyde (PFA) in 1 × PBS at room temperature for 15 min before washing 3 times with 1 × PBS, followed by permeabilization with 0.1% Triton X-100 for 15 min. Then the cells were incubated overnight at 4°C with primary antibody (myelin basic protein [MBP], 1:50, Chemicon, Temecula, CA; NG2, 1:200, Millipore, Etobicoke, Ontario, Canada). Then the cells were incubated with the corresponding fluorescein isothiocyanate- or tetramethylrhodamine-conjugated secondary antibody (1:l00, Jackson ImmunoResearch, West Grove, PA) and Hoechst 33342 (1:1000) for 1 h at room temperature. Images were captured with a fluorescence microscope (DXM1200; Nikon, Tokyo, Japan).

### Immunohistofluorescence Staining

Animals were anesthetized and perfused with 4% PFA. Spinal cord tissue was embedded and sectioned, and post-fixed in PFA overnight at 4°C. Tissue sections were first boiled in 10 mmol/L citrate buffer (pH 6.0) for 20 min at 96°C, then permeabilized with 0.3% Triton X-100 for 30 min and blocked by 5% donkey serum for 1 h. The sections were incubated overnight at 4°C with primary antibody (CC1, 1:100, Millipore; GFAP, 1:200, Sigma; IBA1, 1:200, Abcam, Cambridge, UK), and then with the corresponding secondary antibody for 1 h at room temperature. Last, the samples were examined under a confocal microscope (Leica, Buffalo Grove, IL).

### Western Blot Analysis

After treatment with SA or vehicle for 72 h, cells were homogenized in RIPA buffer (Beyotime, Shanghai, China) supplemented with the protease inhibitor phenylmethylsulfonyl fluoride (Beyotime). Then, cell lysates were subjected to Western blotting with a standard protocol as previously described [18, 19]. The primary antibodies used were anti-MBP (1:500), mTOR (1:3000, CST, Danvers, MA), p-mTOR (1:3000, CST) and the secondary antibodies were horseradish peroxidase conjugated anti-actin (1:50000; Kangchen Biotechnology, Shanghai, China). The primary antibodies were diluted in 1 × TBST and samples were incubated overnight at 4°C. After washing with 1 × TBST, samples were incubated with secondary antibodies for 1 h. The protein bands were analyzed and quantified using Image Lab (Bio-Rad, Hercules, CA).

### BrdU Incorporation and TUNEL Assays

BrdU (5-bromo-2-deoxyuridine, 10 μmol/L; Sigma) was added to the medium and incubated for 6 h to label proliferating cells. After fixation in 4% PFA, cells were rinsed three times with 1 × PBS for 5 min, permeabilized with 0.3% Triton X-100 for 10 min, then incubated in 2 N HC1 for 30 min and neutralized in 0.1 mol/L sodium borate for 25 min. Finally, the cells were incubated with primary anti-BrdU (1:100, Sigma) overnight at 4°C as described for Immunocytofluorescence Staining above.

TUNEL assays were carried out with the *in situ* cell death detection kit, TMR red (12156, Roche, Indianapolis, IN), according to the manufacturer’s instructions. After fixation in 4% PFA, samples were incubated with the TUNEL reaction solution mixture for 1 h at 37°C and then stained with Hoechst 33342 for 5 min at room temperature.

### Histological Staining

The spinal cords were isolated from LPC and EAE mice and cut into continuous paraffin sections (4 μm). For Luxol fast blue (LFB) staining, sections were stained with LFB solution overnight in a humid incubator at 60°C, then rinsed with 95% ethanol for 5 min, 0.05% lithium carbonate, and 70% ethanol for 20 s, then washed with water.

For hematoxylin and eosin (H&E) staining, sections were stained with hematoxylin for 3 min–5 min, then rinsed in ethanol with 1% HCl for 10 s and 1% ammonia water, then counterstained with eosin. After dehydration through a series of graded ethanols and cleared with xylene, the sections were mounted in Permount mounting medium (Fisher Scientific, Pittsburgh, PA).

### Statistical Analyses

Data are presented as mean ± SD or mean ± SEM from at least three independent experiments unless otherwise indicated. One-way ANOVA with Tukey’s *post hoc* test was used for multiple groups and Student’s *t* test for two groups. The EAE model was analyzed using the nonparametric Mann–Whitney *U* test to compare two groups or the Kruskal–Wallis test with Dunn’s *post hoc* test to compare four groups. *P *< 0.05 was considered statistically significant.

## Results

### SA Promotes the Expression of Myelin Basic Protein (MBP) in OPCs *In Vitro*

**Fig. 1:**
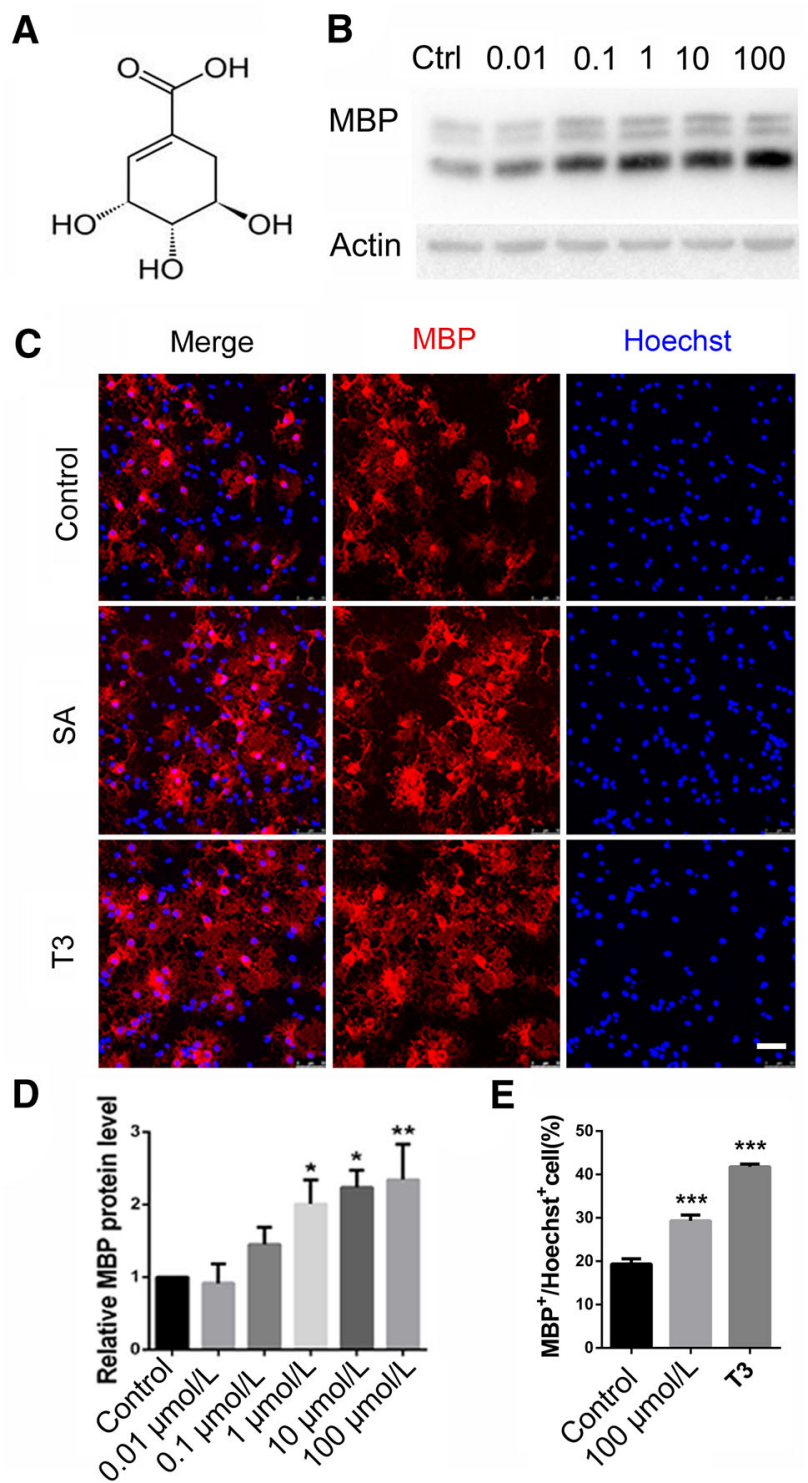
SA increased myelin basic protein (MBP) expression in OPCs *in vitro*. **A** Structural formula of SA. **B** Western blots of MBP expression after 0.01 μg/mL, 0.1 μg/mL, 1 μg/mL, 10 μg/mL, and 100 μg/mL SA treatment in cultured primary OPCs. **C** Representative images of immunofluorescence staining for MBP (red) in vehicle, SA (100 μg/mL), and T3 groups. **D** Quantification of the relative expression levels of MBP protein as in **B**. **E** Numbers of MBP-positive cells as in **C**. Data are shown as mean ± SEM. *P < 0.05, **P < 0.01, ***P < 0.001, one-way ANOVA with Tukey’s *post hoc* test. Scale bar, 50 μm.

**Fig. 2:**
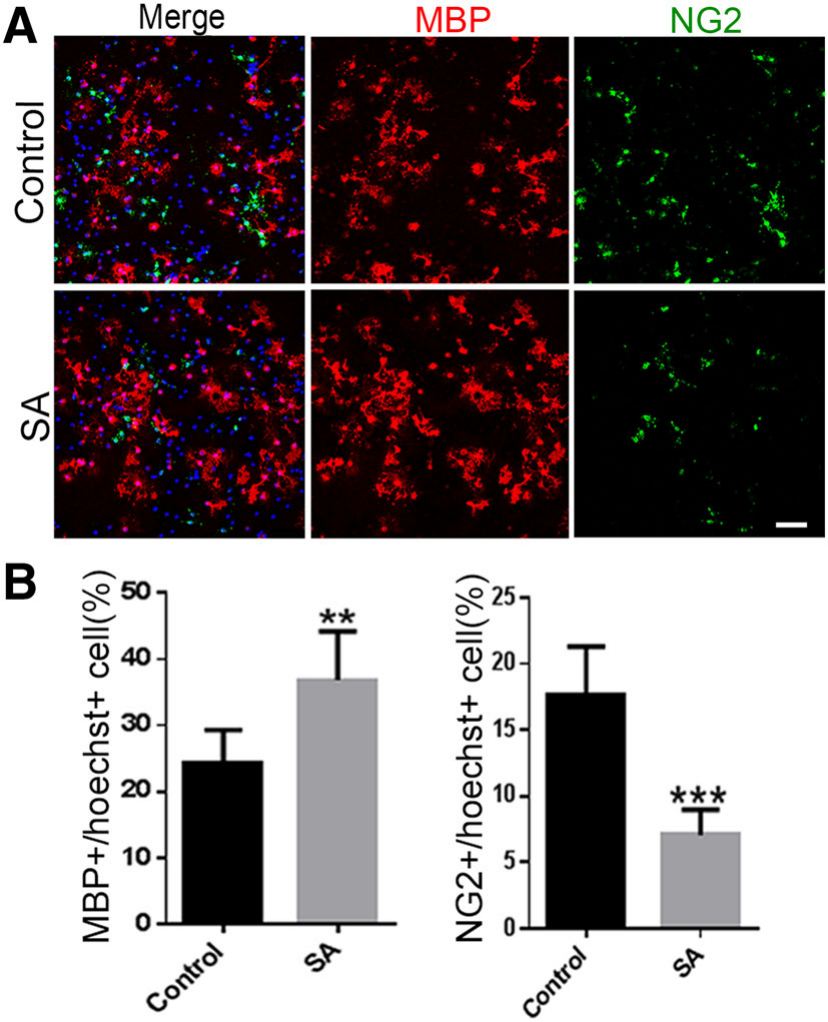
SA decreases the number of NG2-positive cells in OPCs *in vitro*. **A** Representative images of immunofluorescence staining for MBP (red) and NG2 (green) in vehicle and SA (100 μg/mL) groups. **B** Numbers of MBP- and NG2-positive cells as in **A**. Data are shown as mean ± SD. **P < 0.01, ***P < 0.001, Student’s *t*-test. Scale bar, 50 μm.

To unravel the effect of SA on the differentiation of OPCs, we assessed the expression of MBP in cultured OPCs treated with SA (0.01 μg/mL, 0.1 μg/mL, 1 μg/mL, 10 μg/mL, and 100 μg/mL) and vehicle for 72 h. We found that MBP was up-regulated after SA administration (1 μg/mL, 10 μg/mL, and 100 μg/mL), in a dose-dependent manner. The highest level of MBP expression was found in the 100 μg/mL SA treatment group (Fig. 1B, D), showing a 2-fold increase over vehicle-treated cells. Hence, we used 100 μg/mL as the standard concentration for all *in vitro* experiments unless otherwise stated. These results were also confirmed by immunocytochemistry. Three days after SA treatment, the proportion of MBP-positive mature OLs was significantly higher than in the control group (Fig. 1C, E), which was in line with the results obtained with T3 administration as a positive control. To further determine whether SA accelerates the differentiation process from OPCs to mature OLs, we co-stained for NG2 and MBP in SA- and vehicle-treated OPCs. We found that the number of NG2-positive cells was clearly down-regulated while that of MBP-positive cells was up-regulated (Fig. 2A, B). These results revealed that SA could promote the differentiation and maturation of OPCs *in vitro*.

### SA Does not Affect Proliferation and Apoptosis of OPCs

**Fig. 3:**
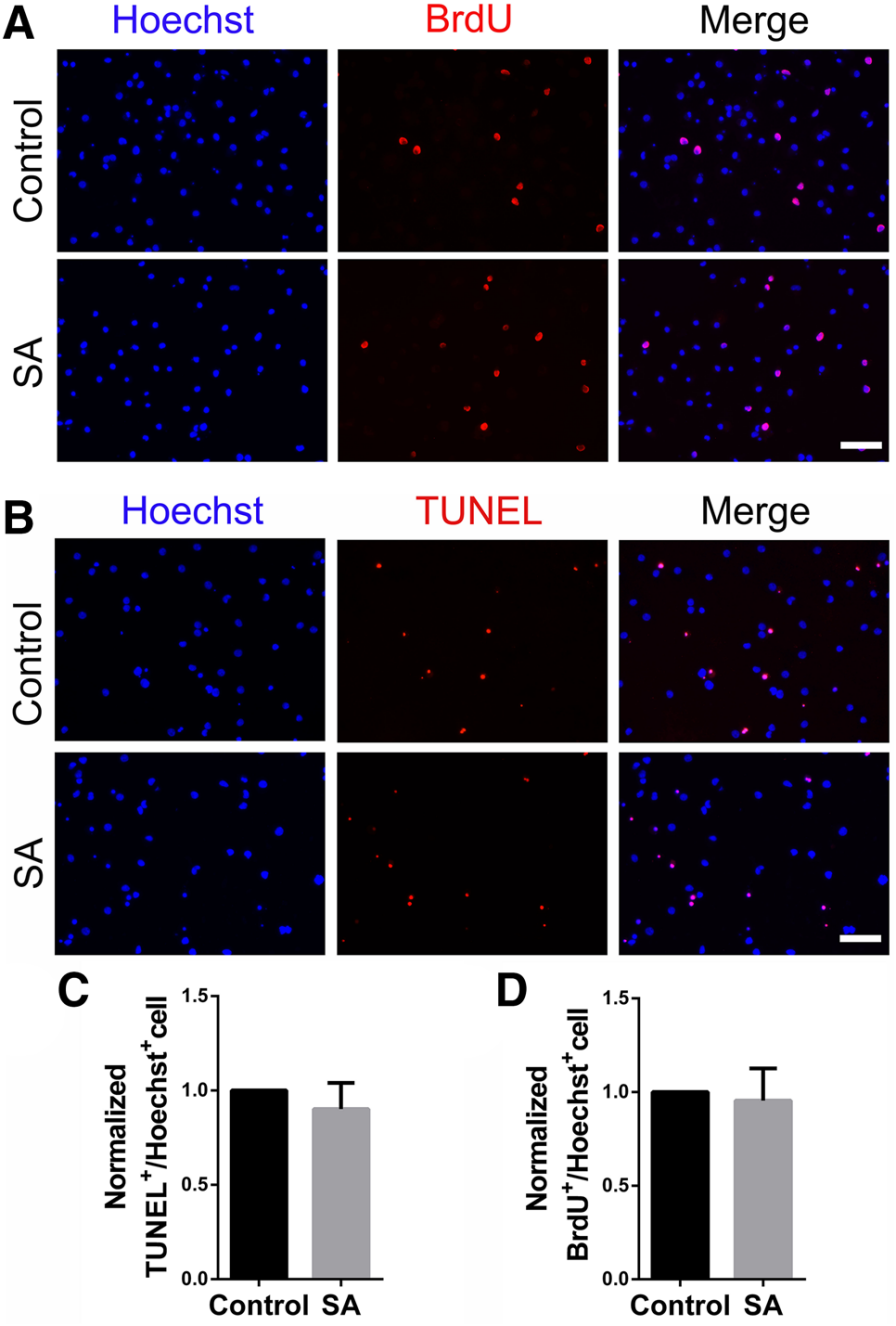
SA does not affect proliferation and apoptosis of OPCs. **A**, **B** Representative images of BrdU-positive proliferating cells (red) (**A**) and TUNEL-positive apoptotic cells (red) (**B**) in SA (100 μg/mL)- and vehicle-treated OPCs. **C, D** Ratios of TUNEL^+^/Hoechst^+^ and BrdU^+^/Hoechst^+^ cells in SA (100 μg/mL)- and vehicle-treated groups. Data are shown as mean ± SD. Student’s *t*-test. Scale bars, 50 μm.

To investigate whether SA regulates the proliferation and apoptosis of OPCs, BrdU incorporation and TUNEL assays were carried out. The results showed no difference in the ratio of BrdU^+^ cells between the SA-treated and control groups (Fig. 3A, D), indicating that SA had no effect on OPC proliferation. In addition, there was no significant difference in TUNEL-positive cells between the SA-treated and control groups (Fig. 3B, C). Taken together, these results suggested that SA specifically promotes the maturation of OPCs without affecting their proliferation and apoptosis.

### SA Alleviates EAE Progression

**Fig. 4:**
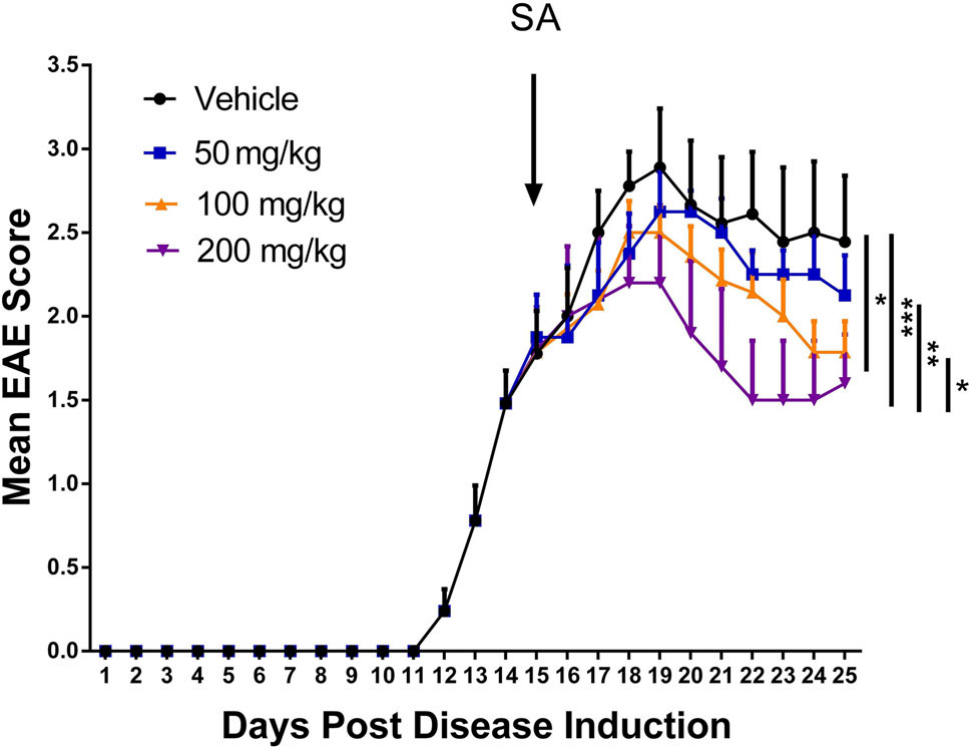
SA ameliorates progression of EAE. The graph shows the daily clinical EAE score in the SA (50 mg/kg, 100 mg/kg, and 200 mg/kg) and vehicle groups (once a day from 15 dpi). Analysis showed significant differences among the groups. Data are shown as mean ± SEM, *n* = 5/group in each experiment; *P < 0.05, **P < 0.01, ***P < 0.001, the nonparametric Mann–Whitney U test was used to compare two groups and the Kruskal–Wallis test with Dunn’s *post hoc* test to compare four groups.

EAE is the most common animal model for demyelinating diseases. Based on the previous finding that SA could promote OPC differentiation *in vitro*, we administered SA to EAE mice to investigate its therapeutic effect. The experimental results showed no significant difference in behavioral score between the low-dose (50 mg/kg SA) and control groups. However, the EAE scores were significantly lower in the 100 and 200 mg/kg SA groups, and the effect of 200 mg/kg SA group was relatively stronger (Fig. 4).

### SA Inhibits CNS Inflammation and Demyelination

**Fig. 5:**
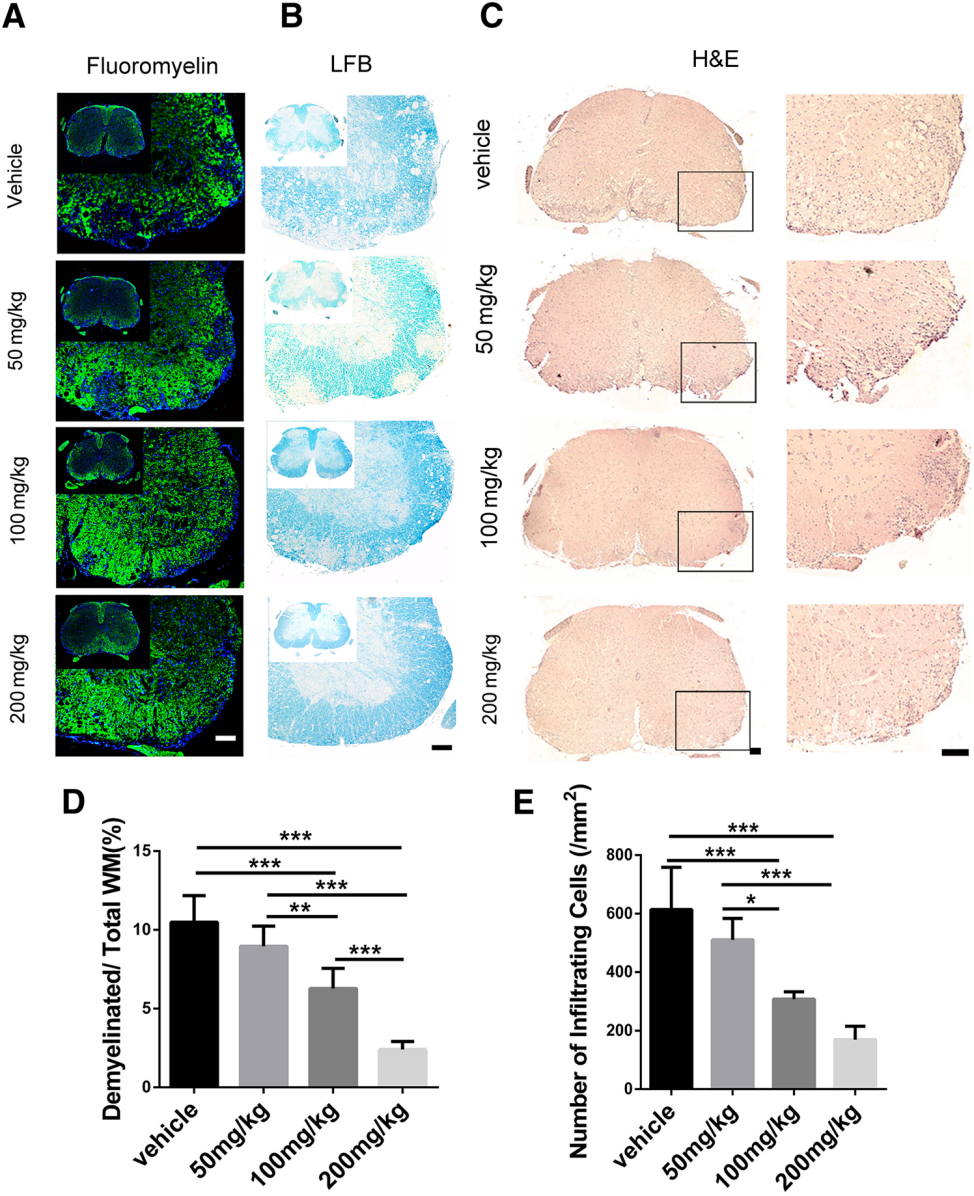
SA inhibits inflammation and demyelination in the CNS of EAE mice. **A**, **B** Representative images of fluoromyelin (**A**) and Luxol fast blue staining (**B**) of spinal cord sections in EAE mice in the SA and vehicle groups at 25 dpi. **C** Representative images of H&E staining showing infiltrating cells in spinal cord sections from EAE mice in the SA and vehicle groups at 25 dpi. **D, E** Quantitative analysis of the demyelination area and number of infiltrating cells as in **B** and **C**. Data are shown as mean ± SEM, *n *= 5/group in each experiment, *P < 0.05, **P < 0.01, ***P < 0.001, one-way ANOVA with Tukey’s *post hoc* test. Scale bars, 100 μm.

**Fig. 6:**
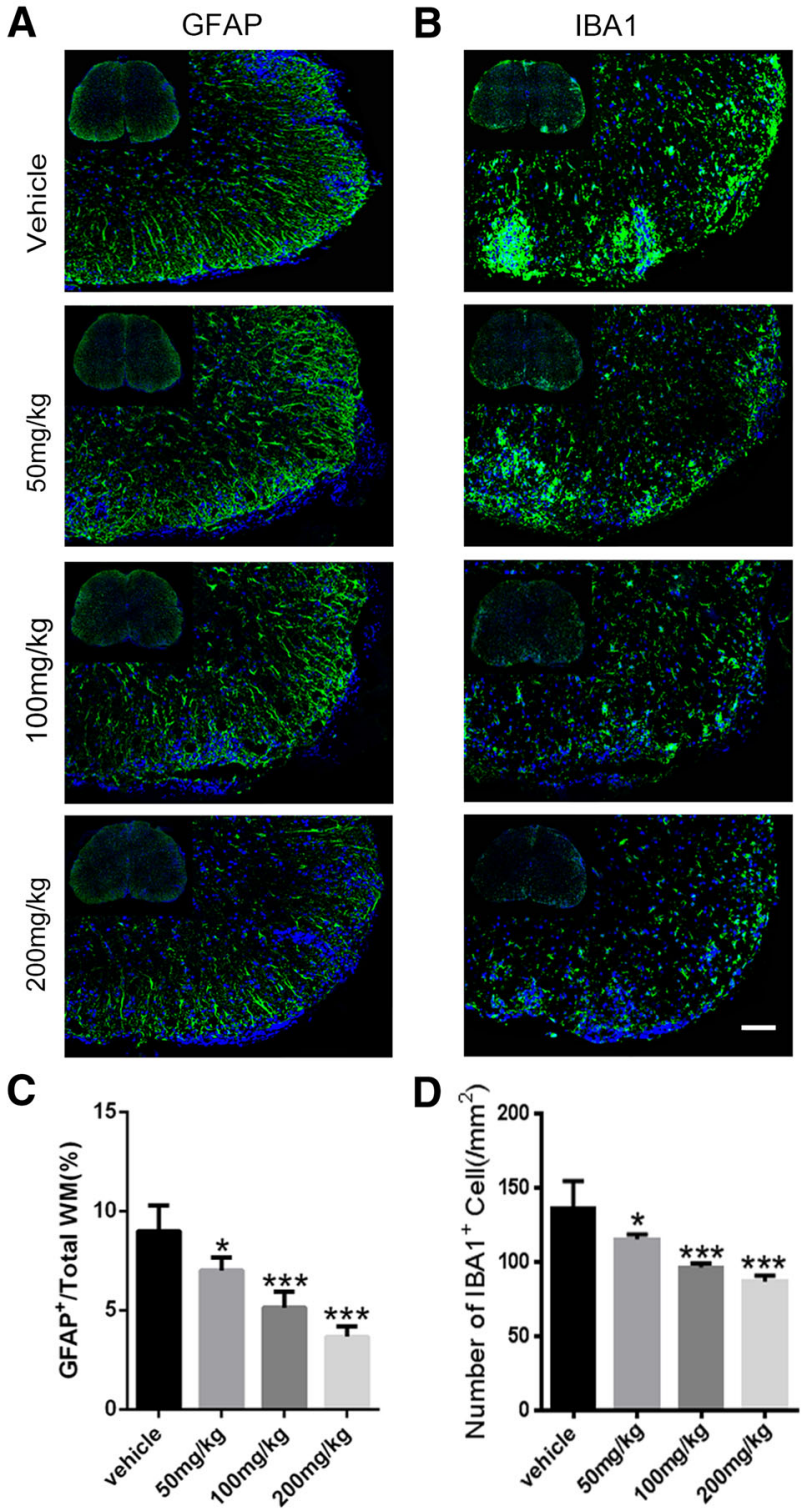
SA downregulates GFAP^+^ and IBA1^+^ cells in the CNS of EAE mice. **A**, **B** Representative immunofluorescence images showing GFAP (green) and IBA1 (green) positive cells in spinal cord sections from EAE mice in the SA and vehicle groups at 25 dpi. **C, D** Numbers of GFAP^+^ and IBA1^+^ cells as in **A** and **B**. Data are shown as mean ± SEM, *n* = 5/group in each experiment, *P < 0.05, ***P < 0.001, one-way ANOVA with Tukey’s *post hoc* test. Scale bar, 100 μm.

Then we used Fluoromyelin, LFB, and H&E staining to examine the spinal cord of EAE mice in the different groups. Fluoromyelin and LFB staining showed no significant difference in the demyelination area between the 50 mg/kg SA and control groups, while that of the 100 mg/kg and 200 mg/kg SA groups was smaller than the control group, with 200 mg/kg SA group displaying the smallest area of demyelination (Fig. 5A, B, D). H&E staining showed no significant difference between the number of inflammatory cells in the 50 mg/kg SA group and that in the control group. The numbers of inflammatory infiltrating cells in the lesions of the 100 mg/kg and 200 mg/kg SA groups were significantly reduced, and the 200 mg/kg group was lower (Fig. 5C, E). Immunofluorescence staining for GFAP and IBA1 (Fig. 6A, B) showed that the numbers of astrocytes and microglia were lower in all SA groups while the effects of 100 mg/kg and 200 mg/kg SA were more apparent (Fig. 6C, D). These results demonstrated that SA could reduce the demyelination and inhibit the infiltration of inflammatory cells in the CNS of EAE mice.

### SA Promotes Remyelination in LPC-Induced Focal Demyelination Model

**Fig. 7:**
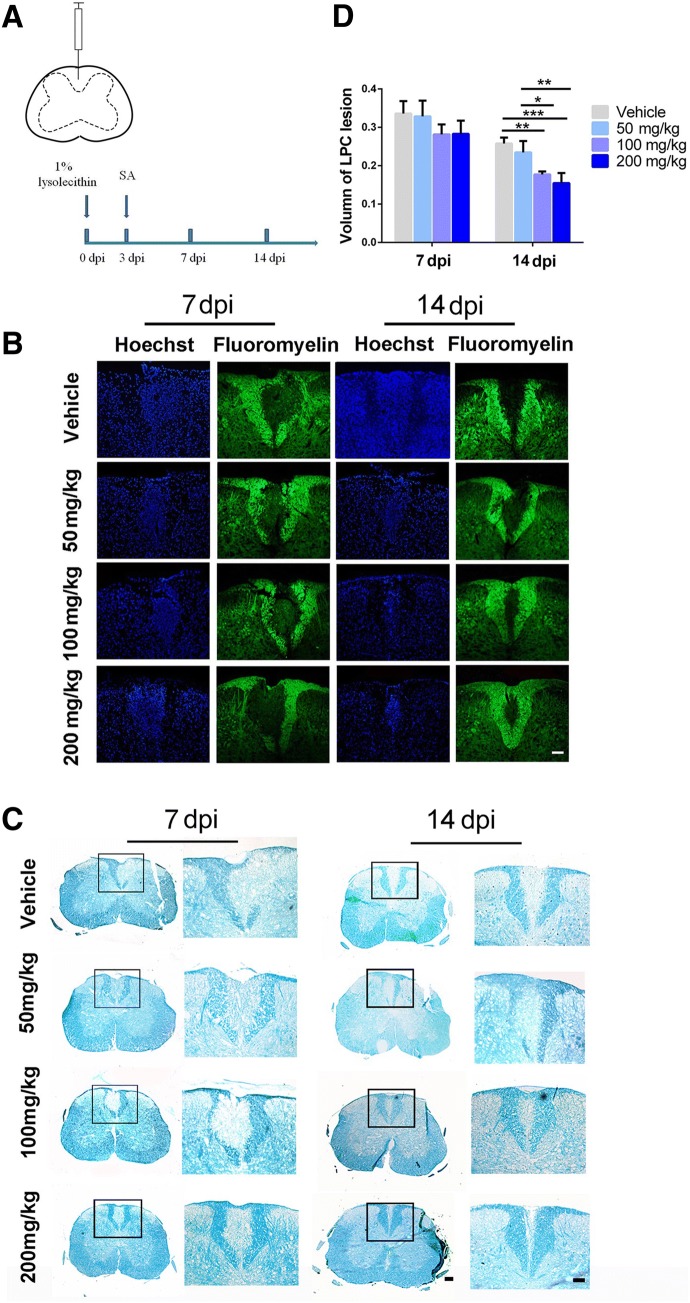
SA enhances remyelination in LPC-induced focal demyelination lesions. **A** Schematic illustrating the injection site and the timing of the LPC model as well as the time SA was administered. **B, C** Representative images of Fluoromyelin (**B**) and LFB staining (**C**) showing the demyelinated region in the dorsal column of the spinal cord. **D** Quantification of the volume of demyelination as in **B** and **C**. Data are shown as the mean ± SEM, *n* = 5/group in each experiment. *P < 0.05, **P < 0.01, ***P < 0.001, one-way ANOVA with Tukey’s *post hoc* test. Scale bars, 100 μm.

**Fig. 8:**
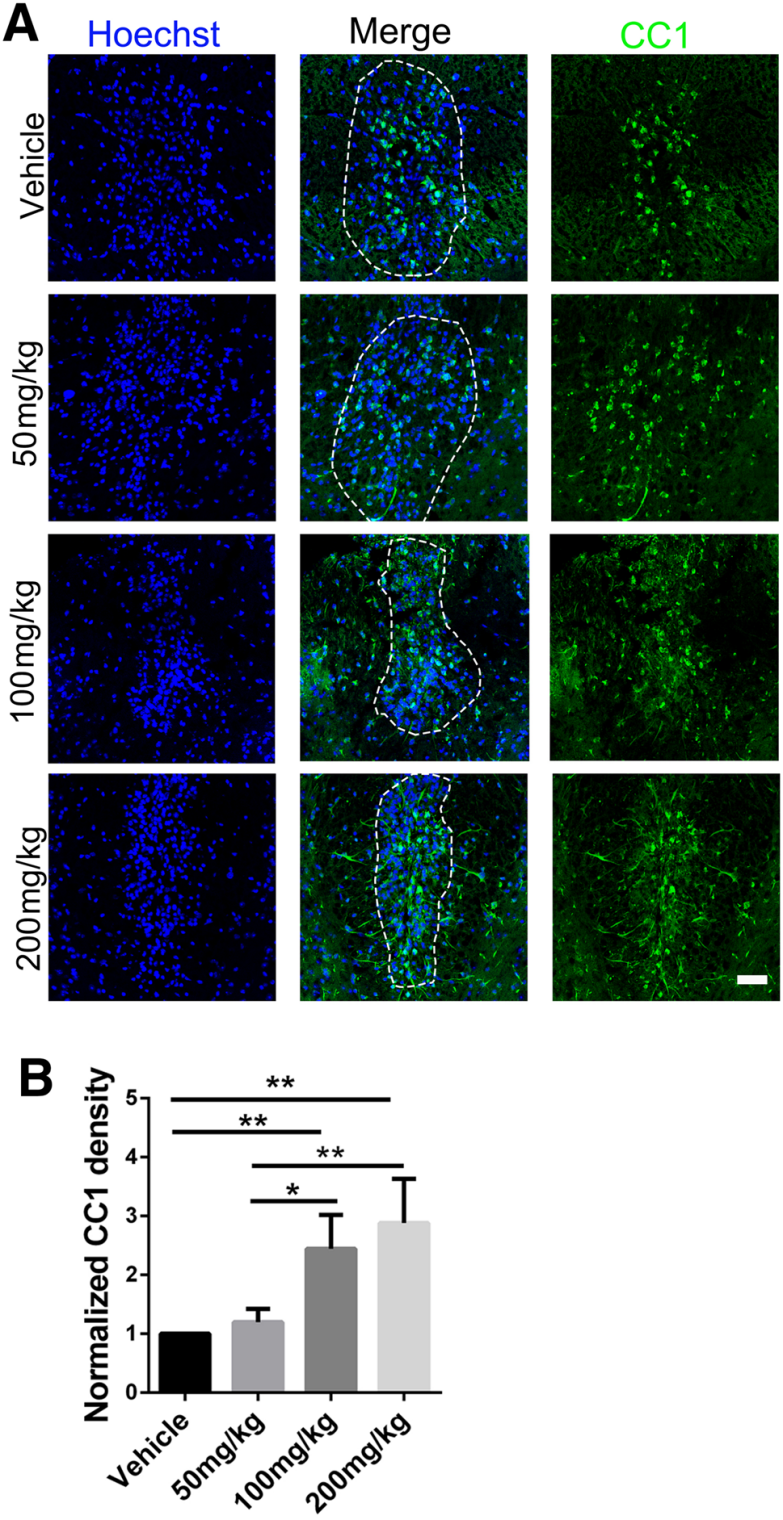
SA increases the number of mature OLs during remyelination in LPC lesions. **A** Representative immunofluorescence images showing CC1 (green) positive cells within the lesion (approximated by dotted line) at 14 dpi in SA (50 mg/kg, 100 mg/kg, and 200 mg/kg) and vehicle-treated mice. **B** Ratios of CC1-positive cells per lesion area as in **A**. Data are shown as mean ± SEM, *n* = 5/group in each experiment, *P < 0.05, **P < 0.01, one-way ANOVA with Tukey’s *post hoc* test. Scale bar, 100 µm.

**Fig. 9:**
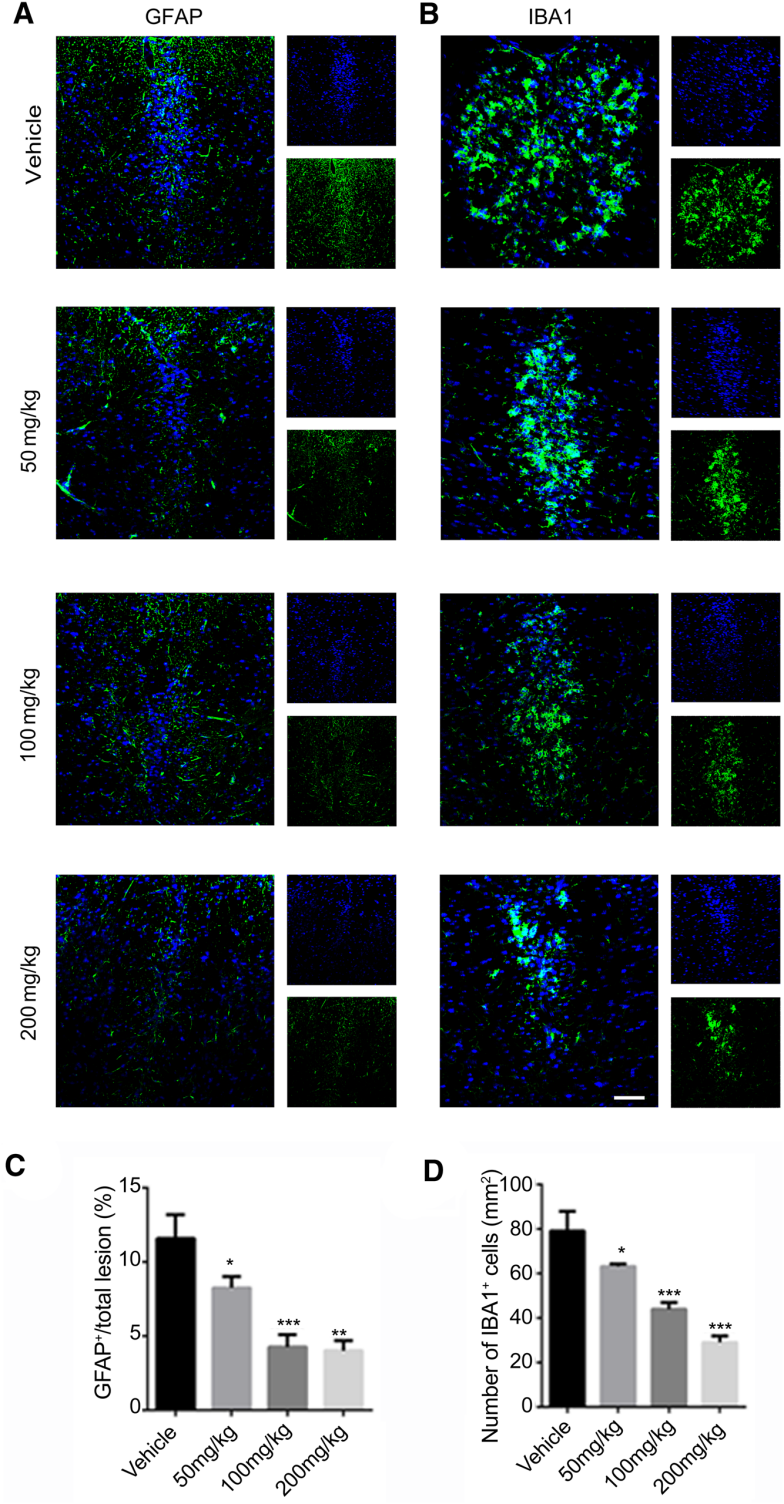
SA reduces the ratio of GFAP^+^ and IBA1^+^ cells during remyelination in LPC lesions. **A**, **B** Representative immunofluorescence images showing GFAP (**A**) and IBA1 (**B**) positive cells within the lesions at 14 dpi in the SA (50 mg/kg, 100 mg/kg, and 200 mg/kg) and vehicle groups. **C, D** Ratios of GFAP (**C**) and IBA1 (**D**) positive cells per lesion area as in **A** and **B**. Data are shown as mean ± SEM, *n* = 5/group in each experiment, *P < 0.05, **P < 0.01, ***P < 0.001, one-way ANOVA with Tukey’s *post hoc* test. Scale bar, 100 µm.

EAE is a chronic demyelinating model induced by inflammation [21]. To clarify the specific impact of SA in promoting OPC differentiation and maturation *in vivo*, we further explored the effect of SA on the LPC-induced focal demyelination model (Fig. 7A), a non-T cell-dependent model [22]. The LPC model shows demyelination at 3 dpi, displays the maximum area of demyelination at 7 dpi, and exhibits clear remyelination at 14 dpi [45, 46]. From 3 dpi, SA or vehicle was administered daily. The results of Fluoromyelin and LFB staining showed no significant difference in the demyelination volume at 7 dpi among the different groups (Fig. 7B, D). However, at 14 dpi, the demyelination volume was significantly decreased in the SA-treated groups (100 mg/kg and 200 mg/kg), suggesting that SA promotes remyelination in the LPC model (Fig. 7C, D). To further verify that the remyelination-promoting effect of SA is due to an increase in newly-generated OLs from OPCs rather than pre-existing OLs, we assessed the numbers of mature OLs by immunofluorescence with CC1 antibody in the different groups. The number of CC1^+^ cells in the center of lesions was significantly higher in the SA groups (100 mg/kg and 200 mg/kg) than in the control group at 14 dpi (Fig. 8A, B). This result was consistent with our previous *in vitro* experiments and suggested that SA does promote the differentiation of OPCs in the LPC-induced focal demyelination lesions. We also found that different doses of SA decreased the numbers of GFAP^+^ astrocytes and IBA1^+^ microglia (Fig. 9).

### SA Promotes the Differentiation of OPCs by Activating the mTOR Signaling Pathway

**Fig. 10:**
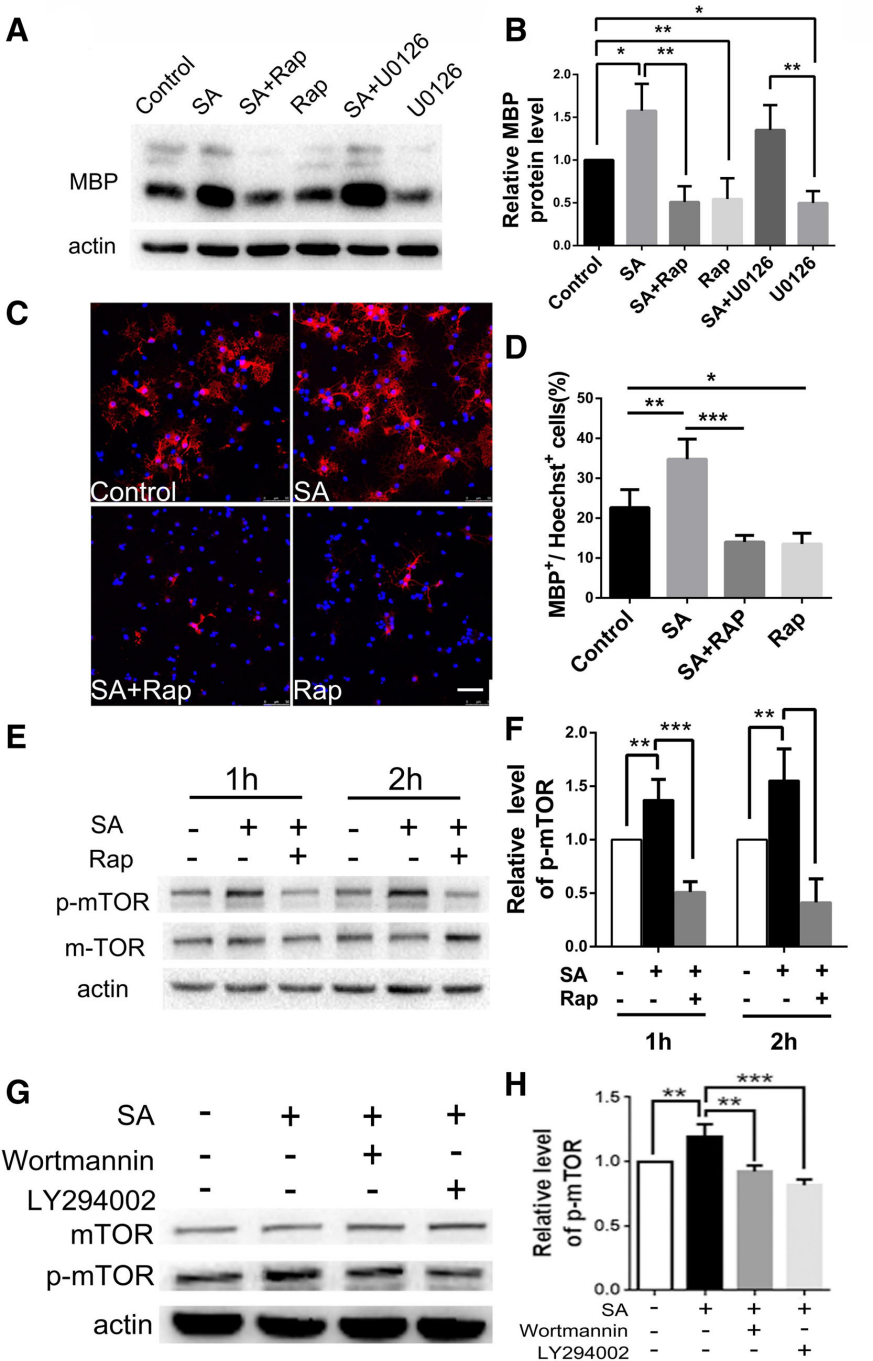
SA promotes OPC differentiation through activating the mTOR pathway. **A** Western blots showing the MBP expression in OPCs induced by SA plus the mTOR inhibitor rapamycin (Rap) (10 nmol/L) or the Erk1/2 inhibitor U0126 (2 nmol/L) and their control groups. **B** Relative expression levels of MBP protein as in **A**. **C** Representative immunofluorescence images showing MBP-positive cells (red) in the SA plus Rap group *versus* its controls. **D** Numbers of MBP-positive cells as in **C**. **E** Western blots showing the p-mTOR levels in the SA plus Rap group *versus* its controls. **F** Relative p-mTOR levels as in **E**. **G** Western blots showing the p-mTOR expression levels in SA plus wortmanin or LY294002 group *versus* its controls. **H** Relative p-mTOR levels as in **G**. Data are shown as mean ± SD. *P < 0.05, **P < 0.01, ***P < 0.001, Student’s *t*-test. Scale bar, 50 μm.

Previous studies have demonstrated that both the Ras/Raf/Mek/Erk and the PI3 K/Akt/mTOR pathways play important roles during OL lineage progression [23–26]. To reveal the pathway targeted by SA, we assessed the MBP expression in OPCs treated with different inhibitors plus SA. Adding rapamycin or U0126 alone to the OPC medium significantly reduced the expression of MBP (Fig. 10A, B), consistent with a previous report [26]. However, rapamycin, an mTOR inhibitor, dramatically blocked the increase of MBP expression in the 100 µg/mL SA group, but the MEK inhibitor U0126 did not (Fig. 10A, B). Meanwhile, the increased MBP-positive cells with SA treatment were diminished after rapamycin was added to the culture (Fig. 10C, D). Moreover, the level of phosphorylated mTOR was significantly elevated after SA treatment compared with the control group (Fig. 10E, F). To verify that the differentiation-promoting effect of SA is mediated by the PI3 K/Akt/mTOR pathway, we incubated OPCs with SA plus Wortmannin and LY294002 which are inhibitors of PI3 K, an up-stream molecule in the Akt/mTOR pathway (Fig. 10G, H). Western blot indicated that both inhibitors of PI3 K reduced the phosphorylation level of mTOR to the level of the vehicle control. Taken together, our results indicated that SA could promote the differentiation of OPCs *via* the PI3 K/Akt/mTOR signaling pathway.

## Discussion

Generally, remyelination proceeds spontaneously in response to CNS demyelination, and this pathophysiological process depends on the differentiation and maturation of OPCs [27]. After demyelination, OPCs rapidly switch from the resting to the activated state, followed by recruitment, proliferation, and differentiation into mature OLs. Then, mature OLs form new myelin sheaths to protect axons and restore signal transmission [4, 28, 29]. Although they remain competent to restore myelin sheaths throughout adulthood, OPCs fail to remyelinate in some demyelinating diseases, such as MS, of which the main pathological features are inflammation-mediated demyelination and multifocal lesions with axonal degeneration [30]. At present, the therapeutic strategy for MS mainly depends on immunosuppression and immunomodulation to reduce the recurrence rate of relapsing-remitting MS, while there is no effective remedy for progressive MS [31, 32]. Previous studies have shown that the failure of remyelination in MS is mainly attributed to the inability of differentiation and maturation of OPCs [6, 33]. Therefore, it is of great importance to seek potential treatments to promote the differentiation and maturation of OPCs.

SA has been shown to protect the neuronal cell line SH-SY5Y against oxidative stimulation which usually occurs under neurodegenerative conditions [9]. In addition, its derivative, 3,4-oxo-isopropylidene-shikimic acid, has been reported to have a similar protective effect for astrocytes and neurons in a rat model of cerebral ischemic injury [34]. However, whether SA has a beneficial impact on OPCs is unknown. In the present study, based on the vital function of myelination along with its specificity as a marker of mature OLs, MBP was selected as a target to test the effects of SA on OPC differentiation. SA showed a significant dose-dependent up-regulation of MBP expression. Consistently, the number of MBP-positive mature OLs marked by immunostaining increased remarkably after SA treatment. Meanwhile, SA reduced the number of NG2-positive OPCs. As SA did not affect the proliferation and apoptosis of OPCs, our results suggested that SA specifically promotes the differentiation and maturation of OPCs.

To directly assess the possible application of SA to demyelinating diseases, we assessed the effects of SA on EAE, the most commonly-used animal model for studying MS. We found that SA significantly inhibited the inflammation and demyelination and reduced the number of astrocytes and microglia in the spinal cord, and the overall neurological functional recovery in EAE mice was improved as well. It has been reported that SA inhibits lipopolysaccharide-induced cellular pro-inflammatory cytokines as well as attenuating mechanical hyperalgesia in mice [8] through inhibiting ERK 1/2 and p38 phosphorylation. We presume that the underlying mechanism of SA in EAE is similar, but this needs further investigation.

Since the pathogenesis of EAE is complex and involves a variety of interactions between the immune and nervous systems [35] and the demyelination and remyelination occur concurrently, it is difficult to distinguish between the reduction of demyelination and the promotion of remyelination [17]. In order to further assess the specific effects of SA on remyelination, we introduced a chemical injury model, that is, LPC-induced focal demyelination. In this model, demyelination and remyelination are generated along a reproducible timeline [36]. Most importantly, the interference of inflammatory factors in EAE can be avoided in the LPC model, which thus can simply and directly reflect the effect of a drug on remyelination [35]. Our data demonstrated that SA did not affect the demyelinating process in the LPC model at 7 dpi, while it did promote the maturation of OPCs together with remyelination at 14 dpi. We also found that the numbers of astrocytes and microglia were decreased after SA treatment in the LPC model; however, since these two types of cells play dual roles in the process of remyelination [37], it is still not clear whether they are supportive or destructive.

Both the Ras/Raf/Mek/Erk and the PI3 K/Akt/mTOR pathways play important roles in OL lineage progression [23–25]. Previous studies have shown that SA can suppress pain and pro-inflammatory factors by inhibiting the phosphorylation of Erk1/2 and p38 [12]. Besides, the Erk1/2 pathway is associated with the survival, proliferation, migration, and differentiation of OPCs and myelination [26, 38]. However, in our study, an inhibitor of MEK, an upstream kinase of Erk, did not block the SA-induced differentiation-promoting effects on OPCs, suggesting that these effects are not associated with the Erk1/2 pathway. Several studies have shown that mTOR, a downstream effector of AKT signaling, is crucial for OPC differentiation and myelination [23, 39, 40]. Rapamycin, an mTOR inhibitor, can repress the differentiation of OPCs as well as the expression of most myelin proteins [26]. Consistently, our experimental results suggested that SA increased the level of phosphorylated mTOR, which could be blocked by rapamycin. And the MBP^+^ OL up-regulation by SA was shown to be antagonized by rapamycin. We also verified that a PI3 K inhibitor lowered the level of phosphorylated mTOR to the blank control level. Interestingly, in our experiments either U0126 or rapamycin alone repressed the maturation of OPCs, but SA plus U0126 did not significantly inhibit the maturation process. A possible explanation for this is that both the Akt/mTOR and Mek/mTOR pathways activate mTORC1 by interacting with the TSC1/TSC2 protein complex [41–43], so SA may activate mTOR and induce OPC differentiation *via* Akt when MEK is blocked by its inhibitor U0126. In the present study, we also found that SA promoted mTOR phosphorylation through the PI3 K pathway, which is in accord with previous research showing that SA activates the PI3 K/Akt pathway in hepatocytes [44]. Thus, the induction of OPC differentiation by SA may mainly be mediated by the PI3 K/Akt/mTOR pathway.

In summary, we found for the first time that SA specifically promoted the differentiation and maturation of OPCs *via* the PI3 K/Akt/mTOR signaling pathway, alleviated the severity of EAE, and accelerated remyelination in the LPC model, suggesting the therapeutic potential of SA for demyelinating diseases.
